# Supplementary material for: Tobacco, electronic nicotine delivery system, nicotine replacement therapy, and cannabinoid use during pregnancy: A descriptive cross-sectional survey
Source: PLoS One. 2025 Sep 25;20(9):e0332961. doi: 10.1371/journal.pone.0332961 (PMC12463284; doi:10.1371/journal.pone.0332961)
Supplement: Attachment — Submitted filename: Completed PONE-D-25-19369_reviewer.pdf [file pone.0332961.s001.pdf]

PLOS ONE

Tobacco, electronic nicotine delivery system, nicotine replacement therapy, and cannabinoid use during pregnancy: A descriptive cross-sectional survey

--Manuscript Draft--

**Manuscript Number:**	PONE-D-25-19369
**Article Type:**	Research Article
**Full Title:**	Tobacco, electronic nicotine delivery system, nicotine replacement therapy, and cannabinoid use during pregnancy: A descriptive cross-sectional survey
**Short Title:**	Tobacco, ENDS, NRT, Cannabinoid Use in Pregnancy
**Corresponding Author:**	Anna Schoeni<br>Universitat Bern Berner Institut fur Hausarztmedizin<br>Basel, SWITZERLAND
**Keywords:**	Tobacco; electronic nicotine delivery system; Nicotine replacement therapy; cannabinoids; pregnancy; smoking cessation counseling
**Abstract:**	Introduction<br>Tobacco smoking is associated with adverse health outcomes for both pregnant women and their offspring. Smoking cessation counseling is an effective method to help women quit smoking. Developing a targeted smoking cessation intervention could benefit those who struggle to quit tobacco and potentially reduce the harm due to any co-occurring tobacco use. Assessing the prevalence of tobacco, electronic nicotine delivery systems (ENDS), nicotine replacement therapy (NRT), and cannabinoid use in pregnancy is key to developing such interventions. Thus, we aimed to assess the prevalence and patterns of tobacco, ENDS, NRT, and cannabinoid use in pregnancy. We further aimed to assess the prevalence of smoking cessation counseling intervention.<br>Materials and Methods<br>We conducted a cross-sectional survey among pregnant women attending regular clinical visits at Spitalzentrum Biel between February and May 2023 (n=262). Frequency and proportion along with 95% confidence intervals (CI) were reported for tobacco, ENDS, NRT, and cannabinoid use in pregnancy.<br>Results<br>Tobacco use was reported among 7.6% (20/262, 95% CI: 4.2%-11.1%) of the included pregnant women. Tobacco cigarettes (conventional or roll-on) were used by 7.3% (19/262, 95% CI: 3.8%-10.7%) of the surveyed pregnant women, with 0.8% (2/262, 95% CI: 0.0%-3.4%) of them reporting use of cigarettes along with ENDS and 0.4% (1/262, 95% CI: 0.0%-3.8%) reporting use of the cigarettes with NRT. Cannabinoid use was reported by 3.8% (10/262, 95% CI: 1.1%-7.0%) of pregnant women and all of them used products with Cannabidiol (CBD) only. Additionally, only 25% (5/20, 95% CI: 10.0%-48.3%) of tobacco users had received smoking cessation counseling intervention.<br>Conclusion<br>The estimated prevalence of tobacco, ENDS, NRT, and cannabinoid use among the pregnant women in this survey was 7.6%, 0.8%, 0.4%, and 3.8% respectively. However, among tobacco users, only one-fourth received smoking cessation counseling intervention.
**Order of Authors:**	Sreemanjari Kandhasamy
	Stéphanie Baggio
	Jérôme Mathis
	Yolanda Mattmann
	Emeline Maisonneuve
	Reto Auer
	Alice Panchaud
	Stephen P Jenkinson

Powered by Editorial Manager® and ProduXion Manager® from Aries Systems Corporation

	Anna Schoeni
**Additional Information:**	
**Question**	**Response**
**Financial Disclosure**<br><br>Enter a financial disclosure statement that describes the sources of funding for the work included in this submission. Review the submission guidelines for detailed requirements. View published research articles from *PLOS ONE* for specific examples.<br><br>This statement is required for submission and **will appear in the published article** if the submission is accepted. Please make sure it is accurate.<br><br>**Funded studies**<br>Enter a statement with the following details:<br>• Initials of the authors who received each award<br>• Grant numbers awarded to each author<br>• The full name of each funder<br>• URL of each funder website<br>• Did the sponsors or funders play any role in the study design, data collection and analysis, decision to publish, or preparation of the manuscript?<br><br>Did you receive funding for this work?	Yes
Please add funding details.<br>as follow-up to "**Financial Disclosure**<br><br>Enter a financial disclosure statement that describes the sources of funding for the work included in this submission. Review the submission guidelines for detailed requirements. View published research articles from *PLOS ONE* for specific examples.<br><br>This statement is required for submission and **will appear in the published article** if the submission is accepted. Please make sure it is accurate.	This work has been supported by an Investigator Initiated Clinical Trial (IICT) grant from the<br>Swiss National Science Foundation (SNSF) IICT_33IC30_173552, the Swiss Tobacco Prevention Fund (TPF) 19.017477, and Swiss Cancer Research (SCR), KFS4744-02-2019 (all 3 to Prof. Reto Auer). The funders had no role in the design of the study; in the collection, analyses, or interpretation of data; in the writing of the manuscript, or in the decision to publish the results.

Powered by Editorial Manager® and ProduXion Manager® from Aries Systems Corporation

**Funded studies**<br>Enter a statement with the following details:<br>• Initials of the authors who received each award<br>• Grant numbers awarded to each author<br>• The full name of each funder<br>• URL of each funder website<br>• Did the sponsors or funders play any role in the study design, data collection and analysis, decision to publish, or preparation of the manuscript?<br><br>Did you receive funding for this work?"	
Please select the country of your main research funder (please select carefully as in some cases this is used in fee calculation).<br>as follow-up to "**Financial Disclosure**<br><br>Enter a financial disclosure statement that describes the sources of funding for the work included in this submission. Review the submission guidelines for detailed requirements. View published research articles from *PLOS ONE* for specific examples.<br><br>This statement is required for submission and **will appear in the published article** if the submission is accepted. Please make sure it is accurate.<br><br>**Funded studies**<br>Enter a statement with the following details:<br>• Initials of the authors who received each award<br>• Grant numbers awarded to each author<br>• The full name of each funder<br>• URL of each funder website<br>• Did the sponsors or funders play any role in the study design, data collection and analysis, decision to publish, or preparation of the manuscript?<br><br>Did you receive funding for this work?"	SWITZERLAND - CH
**Competing Interests**	The authors have declared that no competing interests exist.

Use the instructions below to enter a competing interest statement for this submission. On behalf of all authors, disclose any competing interests that could be perceived to bias this work—acknowledging all financial support and any other relevant financial or non-financial competing interests.<br><br>This statement is required for submission and **will appear in the published article** if the submission is accepted. Please make sure it is accurate and that any funding sources listed in your Funding Information later in the submission form are also declared in your Financial Disclosure statement.<br><br>View published research articles from *PLOS ONE* for specific examples.<br><br>**NO authors have competing interests**<br><br>Enter: *The authors have declared that no competing interests exist.*<br><br>**Authors with competing interests**<br><br>Enter competing interest details beginning with this statement:<br><br>*I have read the journal's policy and the authors of this manuscript have the following competing interests: [insert competing interests here]*<br><br>* typeset	
**Ethics Statement**<br><br>Enter an ethics statement for this submission. This statement is required if the study involved:<br><br>• Human participants<br>• Human specimens or tissue<br>• Vertebrate animals or cephalopods<br>• Vertebrate embryos or tissues	The data for the study were collected from pregnant women coming to Spitalzentrum Biel, a public hospital in Switzerland, between 1st of February and 24th of May 2023. The data was collected in a fully anonymized and irreversible form. Thus, The Ethics Commission of the Canton of Bern, Switzerland, waived the requirement for a formal submission, as the project did not fall under the scope of the Swiss Federal Human Research Act (HRA), Article 2, Paragraph 1, due to the way the data were collected (Req-2022-01416).

Powered by Editorial Manager® and ProduXion Manager® from Aries Systems Corporation

• Field research<br><br>Write "N/A" if the submission does not require an ethics statement.<br><br>General guidance is provided below. Consult the submission guidelines for detailed instructions. **Make sure that all information entered here is included in the Methods section of the manuscript.**	

Powered by Editorial Manager® and ProduXion Manager® from Aries Systems Corporation

**Format for specific study types**<br><br>**Human Subject Research (involving human participants and/or tissue)**<br>• Give the name of the institutional review board or ethics committee that approved the study<br>• Include the approval number and/or a statement indicating approval of this research<br>• Indicate the form of consent obtained (written/oral) or the reason that consent was not obtained (e.g. the data were analyzed anonymously)<br><br>**Animal Research (involving vertebrate animals, embryos or tissues)**<br>• Provide the name of the Institutional Animal Care and Use Committee (IACUC) or other relevant ethics board that reviewed the study protocol, and indicate whether they approved this research or granted a formal waiver of ethical approval<br>• Include an approval number if one was obtained<br>• If the study involved *non-human primates*, add *additional details* about animal welfare and steps taken to ameliorate suffering<br>• If anesthesia, euthanasia, or any kind of animal sacrifice is part of the study, include briefly which substances and/or methods were applied<br><br>**Field Research**<br><br>Include the following details if this study involves the collection of plant, animal, or other materials from a natural setting:<br>• Field permit number<br>• Name of the institution or relevant body that granted permission	
**Data Availability**<br><br>Authors are required to make all data underlying the findings described fully available, without restriction, and from the time of publication. PLOS allows rare exceptions to address legal and ethical concerns. See the PLOS Data Policy and FAQ for detailed information.	No - some restrictions will apply

Powered by Editorial Manager® and ProduXion Manager® from Aries Systems Corporation

A Data Availability Statement describing where the data can be found is required at submission. Your answers to this question constitute the Data Availability Statement and **will be published in the article**, if accepted.<br><br>**Important:** Stating 'data available on request from the author' is not sufficient. If your data are only available upon request, select 'No' for the first question and explain your exceptional situation in the text box.<br><br>Do the authors confirm that all data underlying the findings described in their manuscript are fully available without restriction?	
**Describe where the data may be found in full sentences. If you are copying our sample text, replace any instances of XXX with the appropriate details.**<br><br>• If the data are **held or will be held in a public repository**, include URLs, accession numbers or DOIs. If this information will only be available after acceptance, indicate this by ticking the box below. For example: *All XXX files are available from the XXX database (accession number(s) XXX, XXX.*).<br>• If the data are all contained **within the manuscript and/or Supporting Information files**, enter the following: *All relevant data are within the manuscript and its Supporting Information files.*<br>• If neither of these applies but you are able to provide **details of access elsewhere**, with or without limitations, please do so. For example:<br><br>*Data cannot be shared publicly because of [XXX]. Data are available from the XXX Institutional Data Access / Ethics Committee (contact via XXX) for researchers who meet the criteria for access to confidential data.*<br><br>*The data underlying the results presented in the study are available from (include the name of the third party*	The data used and analyzed in this study may be made available upon reasonable request to the corresponding author.

and contact information or URL).<br>• This text is appropriate if the data are owned by a third party and authors do not have permission to share the data.<br><br>* typeset	
Additional data availability information:	

Powered by Editorial Manager® and ProduXion Manager® from Aries Systems Corporation

Manuscript Click here to access/download;Manuscript;Manuscript_PO_MOD_AS.docx

Original Article

Tobacco, electronic nicotine delivery system, nicotine replacement therapy, and cannabinoid use during pregnancy: A descriptive cross-sectional survey

Sreemanjari Kandhasamy[1,2], Stéphanie Baggio[1,3], Jérôme Mathis[4], Yolanda Mattmann[4], Emeline Maisonneuve[1,5], Reto Auer[1,6], Alice Panchaud [1,7], Stephen P Jenkinson[1,6¶], Anna Schoeni[1,8¶*]

[1]Institute of Primary Health Care (BIHAM), University of Bern, Bern, Switzerland
[2]Graduate School of Health Sciences, University of Bern, Bern, Switzerland
[3]Laboratory of Population Health (#PopHealthLab), University of Fribourg, Fribourg, Switzerland
[4]Department of Obstetrics and Gynecology, Centre Hospitalier Bienne, Bienne, Switzerland
[5]Materno-Fetal and Obstetrics Research Unit, Department "Woman-Mother-Child", Lausanne University Hospital
[6]Center of Primary Care and Public Health (UNISANTÉ), University of Lausanne, Lausanne, Switzerland
[7]Service of Pharmacy, Lausanne University Hospital and University of Lausanne, Lausanne, Switzerland
[8] Center for Child Health Analytics (CCHA), Children's Hospital Central Switzerland, Lucerne Cantonal Hospital, Lucerne, Switzerland

***Corresponding author**

Dr. phil. Anna Schoeni

E-Mail: anna.schoeni@unibe.ch

[¶]These authors contributed equally as the last authors

Short title: Tobacco, ENDS, NRT, Cannabinoid Use in Pregnancy

Abstract

Introduction

Tobacco smoking is associated with adverse health outcomes for both pregnant women and their offspring. Smoking cessation counseling is an effective method to help women quit smoking. Developing a targeted smoking cessation intervention could benefit those who struggle to quit tobacco and potentially reduce the harm due to any co-occurring tobacco use. Assessing the prevalence of tobacco, electronic nicotine delivery systems (ENDS), nicotine replacement therapy (NRT), and cannabinoid use in pregnancy is key to developing such interventions. Thus, we aimed to assess the prevalence and patterns of tobacco, ENDS, NRT, and cannabinoid use in pregnancy. We further aimed to assess the prevalence of smoking cessation counseling intervention.

Materials and Methods

We conducted a cross-sectional survey among pregnant women attending regular clinical visits at Spitalzentrum Biel between February and May 2023 (n=262). Frequency and proportion along with 95% confidence intervals (CI) were reported for tobacco, ENDS, NRT, and cannabinoid use in pregnancy.

Results

Tobacco use was reported among 7.6% (20/262, 95% CI: 4.2%-11.1%) of the included pregnant women. Tobacco cigarettes (conventional or roll-on) were used by 7.3% (19/262, 95% CI: 3.8%-10.7%) of the surveyed pregnant women, with 0.8% (2/262, 95% CI: 0.0%-3.4%) of them reporting use of cigarettes along with ENDS and 0.4% (1/262, 95% CI: 0.0%-3.8%) reporting use of the cigarettes with NRT. Cannabinoid use was reported by 3.8% (10/262, 95% CI: 1.1%-7.0%) of pregnant women and all of them used products with

Cannabidiol (CBD) only. Additionally, only 25% (5/20, 95% CI: 10.0%-48.3%) of tobacco users had received smoking cessation counseling intervention.

Conclusion

The estimated prevalence of tobacco, ENDS, NRT, and cannabinoid use among the pregnant women in this survey was 7.6%, 0.8%, 0.4%, and 3.8% respectively. However, among tobacco users, only one-fourth received smoking cessation counseling intervention.

Introduction

Smoking tobacco in pregnancy is a prevalent, modifiable risk factor for infant mortality and morbidity [1,2]. The global prevalence of smoking during pregnancy was estimated at 1.8%, with Europe reporting a prevalence of 8.1% [3]. In the USA, 5.4% of women who gave birth in 2021 reported smoking while being pregnant [4]. In 2022, 8.3% of women giving birth in Australia admitted to smoking during the first 20 weeks of pregnancy, with 71% of them continuing to smoke throughout their pregnancy [5]. Smoking during pregnancy is associated with an increased risk of several adverse maternal and neonatal outcomes such as placenta previa, placental abruption, premature rupture of membranes, stillbirth, preterm birth, and small for gestational age [6–10]. However, smoking cessation can help reduce these adverse effects [11–13].

Smoking cessation counseling has been beneficial in supporting pregnant women to quit tobacco use, but some might need additional support with smoking cessation aids to quit smoking [14–16]. In addition to nicotine replacement therapy (NRT) and pharmacotherapy (Varenicline or Bupropion), women view electronic nicotine delivery systems (ENDS) as an aid for smoking cessation during pregnancy [17]. Indeed, a randomized controlled trial (RCT) observed ENDS to be more effective when compared to NRT for smoking cessation, showcasing the potential for promising smoking cessation support in pregnant tobacco users [18]. However, there is limited evidence on the safety of using ENDS and NRT in pregnancy [19–23]. Yet, women who find it difficult to quit tobacco continue to use ENDS or NRT throughout pregnancy. In the UK, pregnant smokers struggling to quit tobacco are recommended to use tobacco-free alternatives such as ENDS and NRT as a smoking cessation intervention [16,24]. Notably, ENDS are perceived as a “safer alternative to tobacco” by pregnant women, which has led to a substantial increase in the use of ENDS during pregnancy [19,25]. The Behavioral Risk Factor Surveillance System Survey, a large health survey that

included adult women between 18 and 44 years, was conducted in the USA and observed that ENDS use in pregnancy had almost doubled between 2016 and 2018 [26].

Furthermore, cannabinoids have also been reportedly consumed in pregnancy to treat issues such as nausea, vomiting, increase in body weight, sleep issues, stress, anxiety, depression, post-traumatic stress disorder, and chronic pain [27,28]. The data from the cannabis policy study conducted between 2019 and 2021 on individuals aged 16-65 years in the USA and Canada highlighted that approximately 20% of surveyed pregnant women used CBD-containing products [27]. In 2022, a national survey conducted in the USA on substance use observed that around 7.9% of the pregnant population consumed cannabis (which contained a mixture of THC and CBD) [29]. Cannabinoids like CBD and THC are commonly smoked/vaped and also consumed in the form of oils, edibles, and topical skin preparations [30,31]. When cannabinoids are smoked, they are more likely to be co-used/mixed with tobacco, resulting in an increased risk of adverse neonatal outcomes, thus limiting evidence on the safety of the use of cannabinoids in pregnancy [31–36].

Given these observations, developing personalized, evidence-based, targeted interventions for smoking cessation could potentially provide better support to those struggling to quit/reduce tobacco use and also reduce the harm associated with any co-occurring tobacco use [4,23,26,37–40]. Nonetheless, to develop such targeted interventions, understanding the current prevalence of tobacco, ENDS, NRT, and cannabinoid use among pregnant women is essential. Although nationwide surveys conducted in Switzerland until 2012 indicated that around 10% of women smoked during pregnancy, current evidence on tobacco, ENDS, NRT, and cannabinoid use among pregnant women in Switzerland is limited [41–45]. To bridge this gap, this cross-sectional survey was designed with the primary objective of estimating the prevalence and patterns of tobacco, ENDS, NRT, and cannabinoid use among pregnant women attending regular clinical visits at Spitalzentrum Biel in Switzerland. The secondary objective

was to estimate the intention to quit using tobacco among pregnant users. The survey also aimed to assess the prevalence of smoking cessation counseling intervention.

Materials and Methods

Study design

This was a cross-sectional monocentric survey.

Setting

The data for the study were collected from pregnant women coming to Spitalzentrum Biel, a public hospital in Switzerland, between 1st of February and 24th of May 2023. The data was collected in a fully anonymized and irreversible form. Thus, The Ethics Commission of the Canton of Bern, Switzerland, waived the requirement for a formal submission, as the project did not fall under the scope of the Swiss Federal Human Research Act (HRA), Article 2, Paragraph 1, due to the way the data were collected (Req-2022-01416).

Participants

The participants included in the survey were pregnant women who came in for a regular clinical visit (excluding emergency) at Spitalzentrum Biel between February and May 2023. Each participant was eligible to take the survey once. To ensure this, at the beginning of each survey, participants were questioned if they had already taken part in the survey ("*Befragung bereits bei früherer Visite durchgeführt*" translated as "survey already conducted during the previous visit"). Only those who answered "no" to this question were eligible to continue the survey.

Outcomes

The primary outcome of the study is the frequency and proportion of pregnant women using tobacco products (tobacco cigarettes (conventional cigarettes or roll-on cigarettes) cigars, pipes), tobacco heating systems (like IQOS, Glo™, Ploom), or smokeless tobacco (like snus)

and the use of ENDS, NRT and cannabinoid (cannabidiol only (CBD) or with tetrahydrocannabinol (THC) >1%) products.

The secondary outcomes include frequency and proportion of i. pregnant women intending to reduce/quit/have no change to the current use of tobacco products, ii. pregnant women receiving smoking cessation counseling along with a descriptive summary of suggested alternatives (like NRT, ENDS, pharmacotherapy (Bupropion/Varenicline), and other).

Demographic characteristics

Information on the maternal age and the gestational age (weeks of pregnancy) was collected from the pregnant women at baseline.

Instrument

The information for this survey was collected through a questionnaire. The data were collected by midwives who filled in the questionnaires, and the collected data were irreversibly anonymized. The collected information includes demographic information (such as maternal age and gestational age), information on the use of tobacco, ENDS, and NRT products in the last 7 days, types and frequency of use of these products per day/ week, intent to quit using these products during pregnancy, receiving smoking cessation counseling, and cannabinoid use in last 30 days.

Statistical analysis

Descriptive analysis was conducted to summarize baseline characteristics and to report the primary and secondary outcomes. Means and standard deviations were reported for the maternal age. Median and interquartile range (IQR) were reported for the gestational age of the women. The frequency and proportion, along with 95% confidence intervals (CI), were reported for all the outcomes and were stratified based on the trimester of pregnancy. The 95%

CI was calculated using the multinomCI function in R from the DescTools package. All the analyses were conducted with R version 4.3.2 [46].

Results

Baseline characteristics

For this survey, 337 completely anonymized responses were collected at Spitalzentrum Biel. Among the collected responses, 262 responses were included in the analysis, while 75 incomplete responses were excluded from the analysis. The excluded responses included a "yes" to the question "Befragung bereits bei früherer Visite durchgeführt" which translates to "survey already conducted during the previous visit". The average age of the included pregnant women was 31.35 ±5.11 years. More than half (55.3%,145/262) of the included women were in their third trimester (>28 weeks of pregnancy), and the rest were in their second trimester (between 13-27 weeks of pregnancy). The median gestational age of women included in this survey was 34.9 (IQR: 18.5-37.2) weeks.

Prevalence of use of tobacco, ENDS, NRT, and cannabinoid in pregnancy

In this survey, 7.6% (20/262, 95% CI: 4.2%-11.1%) of the included pregnant women reported use of tobacco products. The tobacco products that were reportedly used in this study included tobacco cigarettes (7.3%, 19/262, 95% CI: 3.8%-10.7%) and tobacco heating systems (0.4%, 1/262, 95% CI: 0.0%-3.8%). Tobacco cigarettes were used along with ENDS in 0.8% (2/262, 95% CI: 0.0-3.4%) and with NRT in 0.4% (1/262, 95% CI: 0.0-3.8%) of the pregnant women in the survey. Cannabinoid use was reported among 3.8% (10/262, 95% CI: 1.1%-7.01%) of the pregnant women. All the cannabinoid product users reported using products containing CBD only, and no one used products with THC>1%. No concomitant use of tobacco and cannabinoids was reported among the pregnant women. The results are presented in Table 1

Table 1. Prevalence of tobacco, electronic nicotine delivery systems, nicotine replacement therapy, and cannabinoid use in pregnancy

Prevalence of tobacco, electronic nicotine delivery systems (ENDS), nicotine replacement therapy (NRT), and cannabinoid use in pregnancy	N=262	Proportion	95% confidence interval (CI)
Non-use of tobacco, ENDS, NRT, and cannabinoids	221	84.4%	80.5%-88.8%
Missing data on use of tobacco, ENDS, NRT, and cannabinoids	11	4.2%	3.8%-8.6%
Tobacco use[a]	20	7.6%	4.2%-11.1%
Tobacco cigarettes (Conventional or roll-on cigarettes)	19	7.3%	3.8%-10.7%
with ENDS	2	0.8%	0.0%-3.4%
with NRT	1	0.4%	0.0%-3.8%
Tobacco heating systems	1	0.4%	0.0%-3.8%
with other products[b]	1	0.4%	0.0%-3.1%
Cannabidiol use (Exclusive cannabidiol (CBD) users)[c]	10	3.8%	1.1%-7.0%

Footnotes

[a]Information on tobacco use was not available for 9 participants and for 1 participant the interview was not possible.

[b]Other products that were not specified in the survey.

[c]Information on cannabinoid use was not available for 10 participants and for 1 participant the conversation on cannabinoid use was not possible during the survey.

Prevalence of use of tobacco, ENDS, NRT, and cannabinoids: stratified by trimester of pregnancy

When stratified by trimester, 10.3% (12/117,95% CI: 5.1%-16.1%) of pregnant women in their second trimester reported using tobacco products while only 5.5% (8/145, 95% CI: 1.4%-9.9%) of the pregnant women in their third trimester reported any such use. Among pregnant women in their second trimester, all tobacco users were tobacco cigarette smokers, with 1.7% (2/117,95% CI:0.0%-7.9%) using tobacco cigarettes along with ENDS and 0.9% (1/117,95% CI:0.0%-7.1%) reporting use of tobacco cigarettes with NRT. In the case of pregnant women in their third trimester, 4.8% (7/145, 95% CI:0.7%-9.1%) of pregnant women reported smoking tobacco cigarettes and 0.7% (1/145, 95% CI:0.0%-4.9%) reported using tobacco heating system. No use of ENDS or NRT was reported among the included pregnant women in their third trimester. Cannabinoid use (CBD only) was reported by 5.1% (6/117,95% CI: 0.1%-9.6%) of pregnant women in their second trimester and by 2.8% (4/145,95% CI: 0.0%-6.9%) in their third trimester. The results are shown in Table 2.

Table 2. Prevalence of tobacco, electronic nicotine delivery systems, nicotine replacement therapy, and cannabinoid use in pregnancy stratified based on trimester of pregnancy

Prevalence of tobacco, electronic nicotine delivery systems (ENDS), nicotine replacement therapy (NRT), and cannabinoid use in pregnancy	Second Trimester (N=117)			Third Trimester (N=145)		
	N	Proportion	95% confidence interval (CI)	N	Proportion	95% confidence interval (CI)
Non-use of tobacco, ENDS, NRT, and cannabinoids	95	81.2%	75.2%-88.5%	126	86.9%	82.1%-92.0%
Missing data on use of tobacco, ENDS, NRT, and cannabinoids	4	3.4%	0.0%-22.7%	7	4.8%	0.0%-9.9%
Tobacco use[a]	12	10.3%	5.1%-16.1%	8	5.5%	1.4%-9.9%
Tobacco cigarettes (Conventional or roll-on cigarettes)	12	10.3%	5.1%-16.1%	7	4.8%	0.7%-9.1%
with ENDS	2	1.7%	0.0%-7.9%	0	-	-
with NRT	1	0.9%	0.0%-7.1%	0	-	-
Tobacco heating systems	0	-	-	1	0.7%	0.0%-4.9%
with other products[b]	0	-	-	1	0.7%	0.0%-4.9%
Cannabidiol use (Exclusive cannabidiol (CBD) users)[c]	6	5.1%	0.1%-9.6%	4	2.8%	0.0%-6.9%

Footnotes

[a]Among the women in their second trimester, three had missing data on tobacco use due to unavailable information. In the third trimester, six women had missing data on tobacco use; for five of them, the information was not available, and for one, the interview could not be conducted.

[b]Other products that were not specified in the survey.

[c]Regarding cannabinoid use, among the participants in their second trimester, data was unavailable for three women, while one could not be interviewed during the survey. For those in their third trimester, seven participants had missing data on cannabinoid use, with no information available for any of them

Frequency and prevalence of use of different types of tobacco products, ENDS, and NRT among pregnant users

Among pregnant women reporting tobacco use, 90.0% (18/20, 95% CI: 85%-100%) reported smoking conventional cigarettes, and 50% of them used less than 5 cigarettes per day. Roll-on cigarettes were used by 5.0% (1/20, 95% CI: 0.0%-19.8%) of the tobacco users and 5.0% (1/20, 95%CI: 0.0%-19.8%) also reported using tobacco heating systems. Among the ENDS and NRT users, 2 (50.0%,95% CI: 25.0%-100.0%) pregnant women reported using ENDS, and 1 (25%, 95% CI: 25.0%-86.0%) pregnant women reported use of NRT. The results are summarized in Tables 3 & 4.

Table 3. Prevalence and Frequency of use of tobacco in pregnancy

Tobacco products	Tobacco Users (N= 20)		Frequency (Number/day) N (%)					
	N (%)	95%Confidence interval (CI)	<5	6-10	11-15	>15	Other	No information
Conventional Cigarette	18(90.0%)	85.0%-100.0%	9(50.0%)	5 (27.8%)	0	1 (5.6%)	0	3 (16.7%)
Roll-on Cigarette	1(5.0%)	0.0%-19.8%	0	1 (100.0%)	0	0	0	0
Tobacco heating system	1(5.0%)	0.0%-19.8%	0	0	0	0	0	1(100.0%)

Table 4. Prevalence and Frequency of use of electronic nicotine delivery systems, nicotine replacement therapy, and other products in pregnancy

Electronic nicotine delivery systems (ENDS), nicotine replacement therapy (NRT), and other products use in pregnancy	Number of participants (N=4)		Frequency (Number/day)/ (ml/day [for liquids]) N (%)					
	N (%)	95%Confidence interval (CI)	<5	6-10	11-15	>15	Other	No information
ENDS users								
ENDS without nicotine	2(50.0%)	25.0%-100.0%	0	2 (100.0%)	0	0	0	0
NRT users								
Inhaler	1(25.0%)	0.0%-86.0%	0	1 (100.0%)	0	0	0	0
Other product users	1(25.0%)	0.0%-86.0%	0	0	0	0	0	1 (100.0%)

Intention to quit/reduce/not change the use of tobacco

On questioning the pregnant women reporting tobacco cigarette use about their intention to quit or reduce their current use, it was observed that 31.6% (6/19,95% CI: 10.5%-54.7%) wanted to quit, 36.8% (7/19,95% CI: 15.8%-60%) wanted to reduce, and 26.3% (5/19,95% CI: 5.3%-49.5%) wanted no change to the current use of tobacco cigarettes. However, one of two ENDS users reported an intention to quit smoking, while the only NRT user also reported her intention to reduce tobacco smoking. The results are presented in Table 5.

Table 5. Preference to quit/change tobacco use: by tobacco, electronic nicotine delivery systems, nicotine replacement therapy, other products users in pregnancy

Preference	Conventional or roll-on cigarette users (N=19)		Tobacco heating system users (N=1)		Electronic nicotine delivery systems (ENDS) users (N=2)		Nicotine replacement therapy (NRT) user(N=1)		Other product user[d] (N=1)	
	N (%)	95% CI	N (%)	95% CI	N (%)	95% CI*	N (%)	95% CI	N (%)	95% CI
Want to quit	6(31.6%)	10.5%-54.7%	0	-	1(50.0%)	50.0%-100.0%	0	-	0	-
Wants to reduce	7(36.8%)	15.8%-60%	0	-	-	-	1(100%)	-	0	-
No change	5(26.3%)	5.3%-49.5%	1 (100.0%)	-	1 (50.0%)	50.0%-100.0%	0	-	1 (100%)	-
Missing[a]	1(5.3%)	0.0%-28.4%	0	-	-	-	0	-	0	-
Interested in a study to switching to ENDS[b]	1(5.3%)	0.0%-20.8%	0	-	0	-	0	-	0	-
Missing[c]	1(5.3%)	0.0%-20.8%	0	-	0	-	0	-	0	-

Footnotes

[a]No information was available for these participants

[b]This variable was only recorded for those who used tobacco [conventional/roll-on cigarette or tobacco heating system (IQOS)]

[c]Only 1 participant had missing information on interest in the study to switch to e-cigarettes.

[d]Other products that were not specified in the survey.

CI: Confidence Interval.

Prevalence of smoking cessation counseling intervention in pregnancy

Only 25% (5/20, 95% CI: 10.0%-48.3%) of those who used tobacco products in pregnancy had received a smoking cessation counseling intervention, and all those who received this intervention reported smoking tobacco cigarettes. Only one among those who received the smoking cessation intervention was recommended to use NRT as an alternative. None received a recommendation for pharmacotherapy. The results are presented in Table 6.

Table 6. Smoking cessation intervention for pregnant tobacco product users and alternatives suggested

Smoking cessation intervention	Tobacco users (N=20)	
	N (%)	95% Confidence Interval (CI)
Received a smoking cessation counseling[a]	5 (25.0%)	10.0% -48.3%
Suggested Alternative		
Nicotine replacement therapy (NRT)	1(5.0%)	0.0-27.8%
Not specified	1(5.0%)	0.0-27.8%
Missing[b]	3 (15.0%)	0.0%-37.8%
Missing[c]	2 (10.0%)	0.0%-33.3%

Footnotes

[a]All tobacco users who received smoking cessation counseling were conventional or roll-on cigarette users.

[b]No information was available for these individuals regarding the alternative suggested

[b]No information was available for these individuals on whether they had received smoking cessation counseling

Discussion

In this cross-sectional survey of 262 pregnant women coming for their regular clinical visits in one center in Switzerland, we found a prevalence of tobacco products at 7.6%, ENDS at 0.8%, NRT at 0.4%, and cannabinoid use at 3.8%.

Previously conducted studies observed the prevalence of tobacco use among pregnant women in Switzerland to vary between 7.0% and 21.0% [47–49]. This is in line with the current study showing a prevalence of 7.6%. The prevalence of tobacco use observed in our study is, however, lower than that observed in the National perinatal survey conducted in France. In the French perinatal survey, 16.6% of women smoked tobacco in their third trimester of pregnancy in 2016, which then decreased to 12.2% in 2021 [50–52]. Additionally, upon stratifying tobacco use by trimester, we observed that the number of tobacco product users in the second trimester was nearly twice as high as in the third trimester. It is, however, not clear if this difference is due to ceasing tobacco use fearing adverse effects during later stages of pregnancy, due to underreporting, or chance [53,54].

While assessing the prevalence of cannabinoid use, none of the pregnant women reported using cannabinoid products with THC>1% but reported use of only CBD products. This could be due to potential underreporting of THC use, as the use of cannabinoid products with THC>1% for recreational purposes is to date prohibited in Switzerland [55]. Additionally, unlike THC, CBD is less psychoactive and thus perceived to be "safe" when compared to THC [35]. Nevertheless, there is a paucity of evidence on the safety of using CBD in pregnancy. Preclinical studies have pointed out potential gender-specific deficits in problem-solving, motor, and discriminatory abilities [56,57]. However, more studies with real-world data are necessary to fully understand the effect of CBD on perinatal outcomes. Like tobacco use, when stratified by trimester, the number of CBD product users in the third trimester was approximately half of that observed in the second trimester. In Switzerland, the use of CBD products containing 3-20% CBD and THC<1% for smoking is increasing [58,59]. A survey conducted among the general population in Switzerland has highlighted the co-use of tobacco, in addition to e-cigarettes and cannabis among CBD users [60]. Although no co-use of these products was observed in our study, this

could still be due to potential underreporting. Additionally, the form or route of consumption of CBD was not collected in this study.

On assessing their intention to quit smoking, 32% of pregnant women in this survey wanted to quit smoking tobacco cigarettes. Observational studies conducted in the past found a significant association between quitting tobacco use during pregnancy and a positive impact on birth weight & neonatal body composition in infants [14,61–64]. A cross-sectional study on live births between 2011 and 2017 observed that women who quit smoking after knowing about pregnancy or during the first trimester had a lower risk of preterm birth compared to those who smoked throughout the pregnancy [65]. Nevertheless, 37% of tobacco cigarette smokers in this survey sample wanted to reduce the use of tobacco in pregnancy. Although quitting tobacco use during pregnancy provides numerous benefits and should be the primary goal, reducing tobacco use can still have positive effects for women who find it difficult to quit. Therefore, they should be encouraged to reduce their tobacco use [14]. Around 26% of pregnant women smoking cigarettes in this survey sample wanted no change in the use of tobacco. Educating this subgroup of pregnant women on the merits of smoking cessation by healthcare providers could be beneficial [66].

In our study, a small proportion of women reported ENDS (0.8%) and NRT (0.4%) use. Among them, one ENDS user mentioned her intent to quit using tobacco, and the sole NRT user also intended to reduce smoking. Additionally, NRT was also suggested as an alternative to support smoking cessation to one pregnant woman who reportedly smoked tobacco and also received smoking cessation counseling in our study. In addition, none were suggested with pharmacotherapy as an alternative. ENDS, NRT, and pharmacotherapy with Varenicline or Bupropion are reportedly used for smoking cessation in pregnancy and can help mitigate the harmful effects associated with tobacco [25,67]. An RCT assessing the impact of the use of NRT in pregnancy observed a significant reduction in the number of smoked cigarettes among

pregnant women and an increase in birth weight and gestational age with NRT use [68]. The Pregnancy trial of E-cigarettes and Patches (PREP) study, a recently conducted RCT, highlighted that the use of ENDS and NRT can improve smoking cessation without increasing the risk of adverse outcomes in pregnancy when compared to abstainers who do not use such products [69]. A recently published retrospective cohort study also observed that the use of NRT, Varenicline, or Bupropion during pregnancy was not associated with an increased risk of major congenital malformations compared to smoking during the first trimester. Although more research is required to assess the effectiveness and safety of NRT and pharmacotherapy use in pregnancy, providing cessation support using these resources could be beneficial in reducing the adverse outcomes due to smoking in pregnancy [14, 70,71].

It is noteworthy that, despite many pregnant tobacco users willing to quit or reduce tobacco use, only 25% of pregnant tobacco users received smoking cessation counseling in our study. This could be due to the potential underreporting of tobacco use in pregnancy to healthcare providers. Underreporting of tobacco use in pregnancy could arise due to the stigma and stereotypes associated with it [53]. The stigma associated with smoking could motivate women to conceal smoking and sometimes encourage them to binge smoke, which could have harmful effects on the mother and fetus [72]. The fact that a considerable proportion of pregnant women smoking cigarettes reported their intentions to quit (32%) or reduce (37%) tobacco use in this anonymous survey highlights the potential need for an intervention that effectively maintains patient anonymity to support smoking cessation efforts. A digital intervention, such as a smartphone app for smoking cessation, could be an effective solution, as it is cost-effective, highly flexible, and preserves patient anonymity [73]. Further studies should be conducted to fully understand the potential for a personalized digital intervention for smoking cessation in pregnant women. In our anonymized survey, 4.2% of included pregnant women still did not provide any response when questioned on their tobacco, ENDS, NRT, and cannabinoid use. In

these cases, using digital intervention to collect such information, while preserving the anonymity of the patient might improve the response rate.

Strengths and limitations

In contrast to previous studies, this study assessed the frequency of use of tobacco, ENDS, NRT, and cannabinoid products stratified by pregnancy trimester. Notably, it is among the few studies that also examined cannabinoid use among pregnant women. Nonetheless, the study has several limitations. This is a single-centered study with a small sample size, and information on the socioeconomic and psychological characteristics of the women included in the study was not collected. Thus, it was not possible to assess if our study cohort was a representative sample of the pregnant population in Switzerland or a high-risk group. Another important limitation is that all substance use has been self-reported, which could also be prone to underreporting, thus resulting in an underestimation of the prevalence of such use in our sample. In the case of cannabinoid use, legal constraints on THC use in Switzerland could also have resulted in the underreporting of THC use in pregnancy, resulting in an underestimation of its prevalence. Also, the form of consumption of CBD was not collected, which limits the knowledge on the potential co-use of tobacco by pregnant women. Thus, the result of this study needs to be interpreted with caution.

Conclusion

This monocentric cross-sectional survey conducted in Switzerland focused on assessing the proportion of tobacco, ENDS, NRT, and cannabinoid use among pregnant women at a hospital for regular check-ups in Biel, Switzerland. The results showed that 7.6% of pregnant women used tobacco products. ENDS and NRT were reportedly used by 0.8% and 0.4% of the pregnant women, and 3.8% of the pregnant women reported the use of cannabinoids, particularly products containing only CBD during pregnancy. Although many pregnant tobacco users were

willing to reduce or quit smoking, only 25% of them received smoking cessation counseling. The use of digital intervention could improve reporting of tobacco use and promote participation in smoking cessation counseling. Future studies should, however, evaluate the potential of such digital strategies. Additionally, further research is required to collect comprehensive information on the form of cannabinoid use among pregnant women in Switzerland to understand the co-use of tobacco among cannabinoid users.

Acknowledgements

We thank all the pregnant women for their participation, all the midwives for their help in collecting the data, and the clinic for their willingness to participate in the survey.

References

1. Bartal M. Health effects of tobacco use and exposure. Monaldi Arch Chest Dis Arch Monaldi Mal Torace. 2001 Dec;56(6):545–54. Available from: https://pubmed.ncbi.nlm.nih.gov/11980288/

2. Crume T. Tobacco Use During Pregnancy. Clin Obstet Gynecol. 2019 Mar ;62(1):128. DOI: 10.1097/GRF.0000000000000413

3. Lange S, Probst C, Rehm J, Popova S. National, regional, and global prevalence of smoking during pregnancy in the general population: a systematic review and meta-analysis. Lancet Glob Health. 2018 Jul;6(7):e769–76. DOI: http://dx.doi.org/10.1016/S2214-109X(18)30223-7

4. Kipling L. Cigarette Smoking Among Pregnant Women During the Perinatal Period: Prevalence and Health Care Provider Inquiries — Pregnancy Risk Assessment Monitoring System, United States, 2021. MMWR Morb Mortal Wkly Rep. 2024;73:393–398. DOI: http://dx.doi.org/10.15585/mmwr.mm7317a2

5. National Core Maternity Indicators, Smoking during pregnancy [Internet]. Australian Institute of Health and Welfare. 2024 [cited 2024 Oct 22]. Available from: https://www.aihw.gov.au/reports/mothers-babies/national-core-maternity-indicators/contents/antenatal-period-indicators/smoking-during-pregnancy

6. Philips EM, Santos S, Trasande L, Aurrekoetxea JJ, Barros H, von Berg A, et al. Changes in parental smoking during pregnancy and risks of adverse birth outcomes and childhood overweight in Europe and North America: An individual participant data meta-analysis of 229,000 singleton births. PLoS Med. 2020 Aug;17(8):e1003182. DOI: 10.1371/journal.pmed.1003182

7. Marufu TC, Ahankari A, Coleman T, Lewis S. Maternal smoking and the risk of still birth: systematic review and meta-analysis. BMC Public Health . 2015 Mar;15(1):239. DOI: https://doi.org/10.1186/s12889-015-1552-5

8. Castles A, Adams EK, Melvin CL, Kelsch C, Boulton ML. Effects of smoking during pregnancy: Five meta-analyses. Am J Prev Med. 1999 Apr;16(3):208–15. DOI: 10.1016/s0749-3797(98)00089-0

9. Zhang Q, Zhang ZC, He XY, Liu ZM, Wei GH, Liu X. Maternal smoking during pregnancy and the risk of congenital urogenital malformations: A systematic review and meta-analysis. Front Pediatr. 2022 Oct;10.DOI: 10.3389/fped.2022.973016

10. Brinchmann BC, Vist GE, Becher R, Grimsrud TK, Elvsaas IKØ, Underland V, et al. Use of Swedish smokeless tobacco during pregnancy: A systematic review of pregnancy and early life health risk. Addict Abingdon Engl. 2023 May;118(5):789–803. DOI: 10.1111/add.16114

11. Bittoun R, Femia G. Smoking cessation in pregnancy. Obstet Med. 2010 Sep;3(3):90–3. DOI: 10.1258/om.2010.090059

12. Bickerstaff M, Beckmann M, Gibbons K, Flenady V. Recent cessation of smoking and its effect on pregnancy outcomes. Aust N Z J Obstet Gynaecol. 2012;52(1):54–8. DOI: https://doi.org/10.1111/j.1479-828X.2011.01387.x

13. Xaverius PK, O'Reilly Z, Li A, Flick LH, Arnold LD. Smoking Cessation and Pregnancy: Timing of Cessation Reduces or Eliminates the Effect on Low Birth Weight. Matern Child Health J. 2019 Oct;23(10):1434–41. DOI: 10.1007/s10995-019-02751-2

14. Ioakeimidis N, Vlachopoulos C, Katsi V, Tousoulis D. Smoking cessation strategies in pregnancy: Current concepts and controversies. Hellenic J Cardiol. 2019 Jan;60(1):11–5. DOI: 10.1016/j.hjc.2018.09.001

15. Vila-Farinas A, Pérez-Rios M, Montes-Martinez A, Ruano-Ravina A, Forray A, Rey-Brandariz J, et al. Effectiveness of smoking cessation interventions among pregnant women: An updated systematic review and meta-analysis. Addict Behav. 2024 Jan;148:107854. DOI: https://doi.org/10.1016/j.addbeh.2023.107854

16. Diamanti A, Papadakis S, Schoretsaniti S, Rovina N, Vivilaki V, Gratziou C, et al. Smoking cessation in pregnancy: An update for maternity care practitioners. Tob Induc Dis. 2019 Aug;17. DOI: https://doi.org/10.18332/tid/109906

17. McCubbin A, Fallin-Bennett A, Barnett J, Ashford K. Perceptions and use of electronic cigarettes in pregnancy. Health Educ Res. 2017 Feb;32(1):22–32. DOI: 10.1093/her/cyw059

18. Hajek P, Przulj D, Pesola F, Griffiths C, Walton R, McRobbie H, et al. Electronic cigarettes versus nicotine patches for smoking cessation in pregnancy: a randomized controlled trial. Nat Med. 2022 May;28(5):958–64. DOI: https://doi.org/10.1038/s41591-022-01808-0

19. Bell L, Whelan M, Thomas L, Edwards E, Lycett D, Hayward K, et al. Use of e-cigarettes in pregnancy: A systematic review of evidence published from 2020-2022. J Public Health. 2023 Jul; DOI: https://doi.org/10.1007/s10389-023-02026-9

20. Vilcassim MJR, Stowe S, Majumder R, Subramaniam A, Sinkey RG. Electronic Cigarette Use during Pregnancy: Is It Harmful? Toxics. 2023 Mar;11(3):278.DOI: 10.3390/toxics11030278

21. Morales-Suárez-Varela M, Puig BM, Kaerlev L, Peraita-Costa I, Perales-Marín A. Safety of Nicotine Replacement Therapy during Pregnancy: A Narrative Review. Int J Environ Res Public Health. 2022 Dec;20(1):250.DOI: 10.3390/ijerph20010250

22. Taylor L, Claire R, Campbell K, Coleman-Haynes T, Leonardi-Bee J, Chamberlain C, et al. Fetal safety of nicotine replacement therapy in pregnancy: systematic review and meta-analysis. Addict Abingdon Engl. 2021 Feb;116(2):239–77. DOI: https://doi.org/10.1111/add.15185

23. Cardenas VM, Fischbach LA, Chowdhury P. The use of electronic nicotine delivery systems during pregnancy and the reproductive outcomes: A systematic review of the literature. Tob Induc Dis. 2019;17:52. DOI: 10.18332/tid/104724

24. McEwen A. Smoking Cessation: A briefing for midwifery staff [internet];2016 [cited 2024 Mar 9]. Available from:

https://www.drugsandalcohol.ie/24129/1/NCSCT_midwifery_briefing_smoking_cessation.pdf

25. Cardenas VM, Ali MM, Fischbach LA, Nembhard WN. Dual use of cigarettes and electronic nicotine delivery systems during pregnancy and the risk of small for gestational age neonates. Ann Epidemiol. 2020 Dec;52:86-92.e2. DOI: https://doi.org/10.1016/j.annepidem.2020.08.002

26. Obisesan OH, Osei AD, Uddin SMI, Dzaye O, Achirica MC, Mirbolouk M, et al. E-Cigarette Use Patterns and High-Risk Behaviors in Pregnancy: Behavioral Risk Factor Surveillance System 2016–2018. Am J Prev Med. 2020 Aug;59(2):187–95.DOI: 10.1016/j.amepre.2020.02.015

27. Bhatia D, Battula S, Mikulich-Gilbertson S, Sakai J, Hammond D. Cannabidiol-Only Product Use in Pregnancy in the United States and Canada: Findings From the International Cannabis Policy Study. Obstet Gynecol. 2022 May;10.1097/AOG.0000000000005603. DOI: 10.1097/AOG.0000000000005603

28. Vanstone M, Panday J, Popoola A, Taneja S, Greyson D, McDonald SD, et al. Pregnant People's Perspectives On Cannabis Use During Pregnancy: A Systematic Review and Integrative Mixed-Methods Research Synthesis. J Midwifery Womens Health. 2022;67(3):354–72. DOI: 10.1111/jmwh.13363

29. Substance Abuse and Mental Health Services Administration.2022 NSDUH Detailed Tables | CBHSQ Data [Internet]; 2022 [cited 2024 Jun 18]. Available from: https://www.samhsa.gov/data/report/2022-nsduh-detailed-tables

30. Barré T, Lahaie E, Di Beo V, Carrieri P, Andler R, Nguyen-Thanh V, et al. Cannabidiol use in France in 2022: Results from a nationwide representative sample of adults. Drug Alcohol Rev. 2024;43(5):1294–304. DOI: https://doi.org/10.1111/dar.13842

31. Young-Wolff KC, Adams SR, Wi S, Weisner C, Conway A. Routes of Cannabis Administration Among Females in the Year Before and During Pregnancy: Results from a Pilot Project. Addict Behav. 2020 Jan;100:106125. DOI: https://doi.org/10.1016/j.addbeh.2019.106125

32. Chu A, Chaiton M, Kaufman P, Goodwin RD, Lin J, Hindocha C, et al. Co-Use, Simultaneous Use, and Mixing of Cannabis and Tobacco: A Cross-National Comparison of Canada and the US by Cannabis Administration Type. Int J Environ Res Public Health. 2023 Mar;20(5). DOI: https://doi.org/10.3390/ijerph20054206

33. Coleman-Cowger VH, Schauer GL, Peters EN. Marijuana and tobacco co-use among a nationally representative sample of US pregnant and non-pregnant women: 2005–2014 National Survey on Drug Use and Health findings. Drug Alcohol Depend. 2017 Aug;177:130–5. DOI: https://doi.org/10.1016/j.drugalcdep.2017.03.025

34. The American college of Obstetricians and Gynecologists. Marijuana Use During Pregnancy and Lactation [Internet]; 2017 [cited 2024 Apr 8]. Available from: https://www.acog.org/clinical/clinical-guidance/committee-opinion/articles/2017/10/marijuana-use-during-pregnancy-and-lactation

35. Sarrafpour S, Urits I, Powell J, Nguyen D, Callan J, Orhurhu V, et al. Considerations and Implications of Cannabidiol Use During Pregnancy. Curr Pain Headache Rep. 2020 Jun];24(7):38. DOI: https://doi.org/10.1007/s11916-020-00872-w

36. Crosland BA, Garg B, Bandoli GE, Mandelbaum AD, Hayer S, Ryan KS, et al. Risk of Adverse Neonatal Outcomes After Combined Prenatal Cannabis and Nicotine Exposure. JAMA Netw Open. 2024 May;7(5):e2410151. DOI: https://doi.org/10.1001/jamanetworkopen.2024.10151

37. Stacey T, Samples J, Leadley C, Akester L, Jenney A. *'I don't need you to criticise me, I need you to support me'.* A qualitative study of women's experiences of and attitudes to smoking cessation during pregnancy. Women Birth. 2022 Nov;35(6):e549–55. DOI: https://doi.org/10.1016/j.wombi.2022.01.010

38. Blanc J, Tosello B, Ekblad MO, Berlin I, Netter A. Nicotine Replacement Therapy during Pregnancy and Child Health Outcomes: A Systematic Review. Int J Environ Res Public Health. 2021 Apr;18(8):4004. DOI: 10.3390/ijerph18084004

39. Milidou I, Henriksen TB, Jensen MS, Olsen J, Søndergaard C. Nicotine Replacement Therapy During Pregnancy and Infantile Colic in the Offspring. Pediatrics. 2012 Mar;129(3):e652–8. DOI: https://doi.org/10.1542/peds.2011-2281

40. Sumodhee D, Walsh H, Brose L, McNeill A, McEwen A, Duaso MJ. Support Provided by Stop-Smoking Practitioners to Co-users of Tobacco and Cannabis: A Qualitative Study. Nicotine Tob Res. 2023 Jul;26(1):23–30. DOI: 10.1093/ntr/ntad115

41. Grob A, Lemola S. Substanzkonsumscreening in der Schwangerschaftsvorsorge. Final report. Bern: Federal Office of Public Health. 2008 May.

42. Grob DA. Substanzkonsum junger Eltern in der Schweiz: im Auftrag des Bundesamtes für Gesundheit, (Verfügung 03.001623) [Internet];2005[cited 2024 Jun 18]. Available from: https://edoc.unibas.ch/23514/1/Substanzkonsum_Eltern_CH.pdf

43. Lemola S, Grob A. Drinking and smoking in pregnancy: what questions do Swiss physicians ask? Swiss Med Wkly. 2007 Jan 27;137(3–4):66–9. DOI: https://doi.org/10.4414/smw.2007.11648

44. Lemola S, Gkiouleka A, Urfer-Maurer N, Grob A, Tritten Schwarz K, Meyer-Leu Y. Midwives' engagement in smoking- and alcohol-prevention in prenatal care before and after the introduction of practice guidelines in Switzerland: comparison of survey findings from 2008 and 2018. BMC Pregnancy Childbirth. 2020 Jan;20:31. DOI: 10.1186/s12884-019-2706-8

45. Bornhauser C, Quack Lötscher K, Seifert B, Simões-Wüst AP. Diet, medication use and drug intake during pregnancy: data from the consecutive Swiss Health Surveys of 2007 and 2012. Swiss Med Wkly. 2017;147:w14572. DOI: 10.4414/smw.2017.14572

46. R Core Team. _R:A Language and Environment for Statistical Computing_. R Foundation for Statistical Computing, Vienna, Austria. 2024 [Internet]. Available from: https://www.R-project.org/

47. Dupraz J, Graff V, Barasche J, Etter J, Boulvain M. Tobacco and alcohol during pregnancy: prevalence and determinants in Geneva in 2008. Swiss Med Wkly. 2013 May; DOI: https://doi.org/10.4414/smw.2013.13795

48. Moussaoui LS, Paillard CN, Delattre M, Desrichard O, Kaiser B. Socio-demographic and motivational correlates of smoking status at term and postpartum. Health Psychol Rep. 2020 Dec;9(3):217–26. DOI: https://doi.org/10.5114/hpr.2020.102135

49. Keller R, Radtke T, Füllemann D, Krebs H, Hornung R. Tabagisme et grossesse. Monitorage sur le tabac – Enquête suisse sur le tabagisme [Tobacco use and pregnancy. Tobacco monitoring Switzerland] [Internet]. 2009 Nov.[Cited 2024 May 21]. Available

from: http://www.stop-tabac.ch/fr/images/stories/documents_stop_tabac/tabagisme%20et%20grossesse%20ofsp.pdf

50. Enquête nationale périnatale 2016. Les naissances et les établissements, situation et évolution depuis 2010 | Direction de la recherche, des études, de l’évaluation et des statistiques [Internet]; 2016 [cited 2024 Oct 22]. Available from: https://drees.solidarites-sante.gouv.fr/publications/rapports/enquete-nationale-perinatale-2016-les-naissances-et-les-etablissements

51. Blondel B, Coulm B, Bonnet C, Goffinet F, Le Ray C. Trends in perinatal health in metropolitan France from 1995 to 2016: Results from the French National Perinatal Surveys. J Gynecol Obstet Hum Reprod. 2017 Dec;46(10):701–13. DOI: https://doi.org/10.1016/j.jogoh.2017.09.002

52. SPF. Enquête nationale périnatale. Rapport 2021. Les naissances, le suivi à deux mois et les établissements [Internet];2021 [cited 2024 Oct 22]. Available from: https://www.santepubliquefrance.fr/import/enquete-nationale-perinatale.-rapport-2021.-les-naissances-le-suivi-a-deux-mois-et-les-etablissements

53. Grant A, Morgan M, Gallagher D, Mannay D. Smoking during pregnancy, stigma and secrets: Visual methods exploration in the UK. Women Birth. 2020 Feb;33(1):70–6. DOI: 10.1016/j.wombi.2018.11.012

54. Tong VT, Althabe F, Alemán A, Johnson CC, Dietz PM, Berrueta M, et al. Accuracy of self-reported smoking cessation during pregnancy. Acta Obstet Gynecol Scand. 2015 Jan;94(1):106–11. DOI: 10.1111/aogs.12532

55. Baltes-Flueckiger L, Steinauer R, Meyer M, Vogel M, Walter M. Effects of cannabis regulation in Switzerland: Study protocol of a randomized controlled trial. Front Psychiatry. 2023 Mar;14:1139325. DOI: 10.3389/fpsyt.2023.1139325

56. Swenson KS, Gomez Wulschner LE, Hoelscher VM, Folts L, Korth KM, Oh WC, et al. Fetal cannabidiol (CBD) exposure alters thermal pain sensitivity, problem-solving, and prefrontal cortex excitability. Mol Psychiatry. 2023 Aug;28(8):3397–413. DOI: 10.1038/s41380-023-02130-y

57. Iezzi D, Caceres-Rodriguez A, Chavis P, Manzoni OJJ. In utero exposure to cannabidiol disrupts select early-life behaviors in a sex-specific manner. Transl Psychiatry. 2022 Dec;12(1):1–13. DOI: https://doi.org/10.1038/s41398-022-02271-8

58. Meier U, Dussy F, Scheurer E, Mercer-Chalmers-Bender K, Hangartner S. Cannabinoid concentrations in blood and urine after smoking cannabidiol joints. Forensic Sci Int. 2018 Oct;291:62–7. DOI: https://doi.org/10.1016/j.forsciint.2018.08.009

59. Bundesamt für Gesundheit BAG, Heilmittelinstitut S Schweizerisches, Bundesamt für Lebensmittelsicherheit und Veterinärwesen BLV, Kantonsapothekervereinigung KAV. Produkte mit Cannabidiol (CBD) und anderen Cannabinoiden, die nicht dem Betäubungsmittelrecht unterliegen: Überblick und Vollzugshilfe [Internet]. 2024 Apr. [Cited 2024 Jul 23]. Available from: https://www.swissmedic.ch/swissmedic/de/home/news/mitteilungen/produkte-mit-cannabidiol--cbd----ueberblick.html

60. Zobel F, Notari L, Schneider E, Rudmann O. Cannabidiol (CBD) : analyse de situation [Internet]. 2018.[Cited 2024 Aug 2]. Available from: https://d-inside.drogoserver.ch/2020/200127_%C3%9Cbersichtsstudie.pdf

61. MacArthur C, Knox EG. Smoking in pregnancy: effects of stopping at different stages. Br J Obstet Gynaecol. 1988 Jun;95(6):551–5. DOI: 10.1111/j.1471-0528.1988.tb09481.x

62. Harrod CS, Reynolds RM, Chasan-Taber L, Fingerlin TE, Glueck DH, Brinton JT, et al. Quantity and timing of maternal prenatal smoking on neonatal body composition: the Healthy Start study. J Pediatr. 2014 Oct;165(4):707–12. DOI: 10.1016/j.jpeds.2014.06.031

63. Lieberman E, Gremy I, Lang JM, Cohen AP. Low birthweight at term and the timing of fetal exposure to maternal smoking. Am J Public Health. 1994 Jul;84(7):1127–31. DOI: 10.2105/ajph.84.7.1127

64. Scherman A, Tolosa JE, McEvoy C. Smoking cessation in pregnancy: a continuing challenge in the United States. Ther Adv Drug Saf. 2018 May;9(8):457–74.DOI: 10.1177/2042098618775366

65. Soneji S, Beltrán-Sánchez H. Association of Maternal Cigarette Smoking and Smoking Cessation With Preterm Birth. JAMA Netw Open. 2019 Apr;2(4):e192514. DOI: https://doi.org/10.1001/jamanetworkopen.2019.2514

66. Fujita ÂTL, Rodrigues-Junior AL, Gomes NC, de Martinis BS, Baddini-Martinez JA. Socio-demographic and psychological features associated with smoking in pregnancy. J Bras Pneumol. 2021;47(5):e20210050. DOI: 10.36416/1806-3756/e20210050

67. Bowker K, Lewis S, Ussher M, Naughton F, Phillips L, Coleman T, et al. Smoking and vaping patterns during pregnancy and the postpartum: A longitudinal UK cohort survey. Addict Behav. 2021 Dec;123:107050. DOI: 10.1016/j.addbeh.2021.107050

68. Oncken C, Dornelas E, Greene J, Sankey H, Glasmann A, Feinn R, et al. Nicotine gum for pregnant smokers: a randomized controlled trial. Obstet Gynecol. 2008 Oct;112(4):859–67. DOI: 10.1097/AOG.0b013e318187e1ec

69. Pesola F, Smith KM, Phillips-Waller A, Przulj D, Griffiths C, Walton R, et al. Safety of e-cigarettes and nicotine patches as stop-smoking aids in pregnancy: Secondary analysis of the Pregnancy Trial of E-cigarettes and Patches (PREP) randomized controlled trial. Addiction. 2024;119(5):875–84. DOI: https://doi.org/10.1111/add.16422

70. Tran DT, Cohen JM, Donald S, Cesta CE, Furu K, Parkin L, et al. Risk of Major Congenital Malformations Following Prenatal Exposure to Smoking Cessation Medicines. JAMA Intern Med. 2025; DOI: https://doi.org/10.1001/jamainternmed.2025.0290

71. Coleman T, Orton S. Using Pharmacologic Smoking Cessation Treatments in Pregnancy. JAMA Intern Med. 2025; DOI: https://doi.org/10.1001/jamainternmed.2025.0278

72. David JC, Fonte D, Sutter-Dallay AL, Auriacombe M, Serre F, Rascle N, et al. The stigma of smoking among women: A systematic review. Soc Sci Med. 2024;340:116491. DOI: https://doi.org/10.1016/j.socscimed.2023.116491

73. Griffiths SE, Parsons J, Naughton F, Fulton EA, Tombor I, Brown KE. Are digital interventions for smoking cessation in pregnancy effective? A systematic review and meta-analysis. Health Psychol Rev. 2018;12(4):333–56. DOI: https://doi.org/10.1080/17437199.2018.1488602
