# Supplementary material for: Tobacco, electronic nicotine delivery system, nicotine replacement therapy, and cannabinoid use during pregnancy: A descriptive cross-sectional survey
Source: PLoS One. 2025 Sep 25;20(9):e0332961. doi: 10.1371/journal.pone.0332961 (PMC12463284; doi:10.1371/journal.pone.0332961)
Supplement: Attachment — Submitted filename: Response to Reviewers.pdf [file pone.0332961.s002.pdf]

REVIEWER COMMENTS AND RESPONSES

I. **Reviewer #1**: Reviewed the article- "Tobacco, electronic nicotine delivery system, nicotine replacement therapy, and cannabinoid use during pregnancy: A descriptive cross-sectional survey." Attached are the suggested edits

a. Would be interesting to know what factors led to this difference in tobacco use between France and Switzerland.

   Response: We thank reviewer for this insight. The higher prevalence of smoking in France could be attributed to the strong socio-cultural legacy of tobacco use in France. Nevertheless, we need more studies that compare and contrast socio cultural, and economical factors to help us understand the observed difference in the prevalence of tobacco use between two countries to the fullest extent. We have now added this information in the manuscript as follows:

   In the manuscript [Pg.17]

   However, the observed difference could stem from the social and cultural differences around smoking in both the countries, particularly with the strong cultural legacy of smoking in France [53]. Nevertheless, more studies are necessary to understand how various social, cultural, and economic factors influence the observed disparity in smoking prevalence between France and Switzerland to the fullest extent.

b. Great point to bring up in the city especially when patients question the safety of NRT compared to nicotine use in pregnancy

   Response: We thank the reviewer for this input.

c. Any information on the risks of using ENDS during pregnancy on the developing fetus ?

   Response: Pesola et al., 2024 observed that there was no difference in the adverse neonatal outcomes such as low birth weight, and NICU (neonatal intensive care unit) admission between children of those who abstained from smoking without using any nicotine products (ENDS, NRT), compared to abstainers who used nicotine products (ENDS, NRT) while being pregnant. Additionally, the study also observed that there was increased risk of preterm birth among those who abstained and not use any nicotine products compared to those who abstained and did use ENDS or NRT during pregnancy.
   This information has now been added to the manuscript as follows:

In the manuscript [Pg.20]

This study also observed no difference in the risk of adverse neonatal outcomes such as low birth weight, and NICU (neonatal intensive care unit) admission between abstainers using ENDS or NRT during pregnancy and abstainers using no such aids [73]. Additionally, abstainers using ENDS or NRT had 78% reduced risk of preterm birth compared to abstainers using no aids, during pregnancy [73]. Although ENDS and NRT are seen to have similar safety profile in pregnancy, an RCT conducted in England observed, that ENDS does not increase the risk of adverse birth outcomes when compared to NRT and also reduced risk of low birth weight by 35% when compared to those using NRT in pregnancy [18]

d. This is a major limitations, as self-report in patients with SUD's is inaccurate in a majority of patients., especially if the reported substance is illegal or has legal implications in the country. However it's tough to avoid this bias as how else would you accurately confirm the amounts ?

Response: We understand and agree with the reviewer's concern. It is indeed difficult to avoid this bias. Despite using an anonymous survey in our study, stigma associated with tobacco and cannabinoid use in pregnancy could have resulted in underreporting of use of these products among those included in the study. . Future studies can try incorporating laboratory test in addition to self-reported use, to validate the anonymized self-report in accurately identifying the users. Nevertheless, use of such test depends on cost, feasibility, and patient's acceptance to participate in such laboratory tests. This has now been incorporated into the manuscript as follows:

In the manuscript [Pg.22]:

Another important limitation is that tobacco and cannabinoid use in this survey has been self-reported which could also be prone to underreporting, particularly on THC use due to legal concerns, thus resulting in an underestimation of the prevalence of such use in our sample. Future studies could incorporate additional laboratory tests to validate the anonymized self-report, based on the cost and feasibility of such tests, to accurately identify the users.

II. **Reviewer #2**: The study "Tobacco, electronic nicotine delivery system, nicotine replacement therapy, and cannabinoid use during pregnancy: A descriptive cross-sectional survey" addresses substance use during pregnancy—a critical concern given the risks to maternal and fetal health—and expands to include CBD, ENDS, and NRT. The

survey captures the self-reported intention to quit or reduce tobacco use, which is useful for intervention development.

Please add these limitations to the study:
a. Key predictors of substance use and cessation (education, employment, relationship status, mental health) were not collected, limiting context and interpretation.
b. A snapshot design limits understanding of whether women quit later in pregnancy or switched substances—insights useful for tailoring interventions.
c. The mode of use (e.g., vaping vs. oral vs. topical) affects fetal exposure risk, but this was not assessed.

Response: We thank reviewer for this valuable input. We have now added these limitations to the study as follows:

In the manuscript [Pg.]
This is a single-centered study with a small sample size, and key information on predictors of substance use and cessation such as education, employment status, relationship statis, the socioeconomic and psychological characteristics of the women included in the study was not collected. Thus, it was not possible to assess if our study cohort was a representative sample of the pregnant population in Switzerland or a high-risk group. Due to the cross-sectional design of the study, it was not possible to collect information if tobacco users tend to quit tobacco or switch to using ENDS/NRT over time in pregnancy. Another important limitation is that tobacco and cannabinoid use in this survey has been self-reported, which could also be prone to underreporting, thus resulting in an underestimation of the prevalence of such use in our sample. Future studies could incorporate additional laboratory tests to validate the anonymized self-report, based on the cost and feasibility of such tests, to accurately identify the users. In the case of cannabinoid use, legal constraints on THC use in Switzerland could also have resulted in the underreporting of THC use in pregnancy, resulting in an underestimation of its prevalence. Also, the mode of consumption (vaping/topical/oral) of CBD was not collected, which limits the knowledge on the potential co-use of tobacco by pregnant women. Thus, the result of this study needs to be interpreted with caution.

d. While missing responses are reported, the potential bias from non-response (4.2% on substance use) is not analyzed or statistically accounted for. Please explain.

Response: We thank the reviewer for this insight. We did observe that 4.2% of participants did not answer questions on the tobacco, ENDS, CBD, or NRT use in pregnancy. However, these participants did provide data on age and gestational week, and were therefore retained in the analysis to reflect the presence of individuals who may be reluctant to disclose sensitive behaviors. To assess the potential impact of non-response on our estimates, we conducted a sensitivity analysis assuming that all non-respondents reported tobacco use during pregnancy. Under this assumption, the prevalence of tobacco use among pregnant women in our sample would increase to 11.8%. This suggests a potential underestimation in our primary prevalence estimates due to non-response.

In the manuscript [Pg. 21]:
In our anonymized survey, 4.2% of included pregnant women still did not provide any response when questioned on their tobacco, ENDS, NRT, and cannabinoid use. However, these participants had provided data on age and gestational week and were therefore retained in the analysis to reflect the presence of individuals who may be reluctant to disclose sensitive behaviors. To assess the potential impact of non-response on our estimates, we conducted a sensitivity analysis assuming that all non-respondents reported tobacco use during pregnancy. Under this assumption, the prevalence of tobacco use among pregnant women in our sample would increase to 11.8%. This suggests a potential underestimation in our primary prevalence estimates due to non-response. Nevertheless, this estimate from sensitivity analysis is still within the prevalence range of 7.0%-21.0%, reported from previous studies estimating prevalence of smoking among pregnant women in Switzerland and lower than that observed in the French perinatal survey. Using digital intervention to collect information on tobacco and cannabinoid use, while preserving the anonymity of the patient might improve the response rate.

III. Journal Requirements

When submitting your revision, we need you to address these additional requirements.

a. Please ensure that your manuscript meets PLOS ONE's style requirements, including those for file naming. The PLOS ONE style templates can be found at https://journals.plos.org/plosone/s/file?id=wjVg/PLOSOne_formatting_sample_main_body.pdf

and https://journals.plos.org/plosone/s/file?id=ba62/PLOSOne_formatting_sample_title_authors_affiliations.pdf

Response: The manuscript has now been formatted based on the guidelines.

b. PLOS requires an ORCID iD for the corresponding author in Editorial Manager on papers submitted after December 6th, 2016. Please ensure that you have an ORCID iD and that it is validated in Editorial Manager. To do this, go to ‘Update my Information’ (in the upper left-hand corner of the main menu), and click on the Fetch/Validate link next to the ORCID field. This will take you to the ORCID site and allow you to create a new iD or authenticate a pre-existing iD in Editorial Manager.

c.i. Please provide additional details regarding participant consent. In the ethics statement in the Methods and online submission information, please ensure that you have specified (1) whether consent was informed and (2) what type you obtained (for instance, written or verbal, and if verbal, how it was documented and witnessed). If your study included minors, state whether you obtained consent from parents or guardians. If the need for consent was waived by the ethics committee, please include this information.

If you are reporting a retrospective study of medical records or archived samples, please ensure that you have discussed whether all data were fully anonymized before you accessed them and/or whether the IRB or ethics committee waived the requirement for informed consent. If patients provided informed written consent to have data from their medical records used in research, please include this information

Once you have amended this/these statement(s) in the Methods section of the manuscript, please add the same text to the “Ethics Statement” field of the submission form (via “Edit Submission”).

For additional information about PLOS ONE ethical requirements for human subjects research, please refer to http://journals.plos.org/plosone/s/submission-guidelines#loc-human-subjects-research.

c.ii. Your ethics statement should only appear in the Methods section of your manuscript. If your ethics statement is written in any section besides the Methods, please delete it from any other section.

Response: Thank you for this input. The ethics statement has now been revised to include information on the informed consent and is presented only in the Methods section under the subheading "Setting" as follows**:**

In the manuscript [Pg.no:6]

The data for the study were collected from pregnant women coming to Spitalzentrum Biel, a public hospital in Switzerland, between February and May 2023. The data used in this study were de-identified and completely anonymized. Thus, the ethics committee of the canton of Bern waived the requirement for a formal submission, as the project did not fall under the scope of the Swiss Federal Human Research Act (HRA), Article 2, Paragraph 1 (Req-2022-01416).Written informed consent was not required due to collection of anonymized data under HRA. However, mid-wives explained the study to the participants and only collected data from those who had provided verbal consent to participate in the survey.

d. We note that the grant information you provided in the 'Funding Information' and 'Financial Disclosure' sections do not match.

When you resubmit, please ensure that you provide the correct grant numbers for the awards you received for your study in the 'Funding Information' section.

Response: Thanks again for this input. The «Financial disclosure» is now presented as follows:

In the online Portal :
This work was supported by an Investigator Initiated Clinical Trial (IICT) grant from the Swiss National Science Foundation (SNSF) (grant number: IICT_33IC30_173552), the Swiss Tobacco Prevention Fund (TPF) (grant number :19.017477), and Swiss Cancer Research (SCR), (grant number: KFS4744-02-2019). RA received all the above mentioned grants. The funders had no role in study design, data collection and analysis, decision to publish, or preparation of the manuscript.

e. i. In the online submission form, you indicated that the data used and analyzed in this study may be made available upon reasonable request to the corresponding author.

All PLOS journals now require all data underlying the findings described in their manuscript to be freely available to other researchers, either 1. In a public repository, 2. Within the manuscript itself, or 3. Uploaded as supplementary information. This policy applies to all data except where public deposition would breach compliance with the protocol approved by your research ethics board. If your data cannot be made

publicly available for ethical or legal reasons (e.g., public availability would compromise patient privacy), please explain your reasons on resubmission and your exemption request will be escalated for approval.

e.ii. We note that you have indicated that there are restrictions to data sharing for this study. For studies involving human research participant data or other sensitive data, we encourage authors to share de-identified or anonymized data. However, when data cannot be publicly shared for ethical reasons, we allow authors to make their data sets available upon request. For information on unacceptable data access restrictions, please see http://journals.plos.org/plosone/s/data-availability#loc-unacceptable-data-access-restrictions.

Before we proceed with your manuscript, please address the following prompts:

- If there are ethical or legal restrictions on sharing a de-identified data set, please explain them in detail (e.g., data contain potentially identifying or sensitive patient information, data are owned by a third-party organization, etc.) and who has imposed them (e.g., a Research Ethics Committee or Institutional Review Board, etc.). Please also provide contact information for a data access committee, ethics committee, or other institutional body to which data requests may be sent.
- If there are no restrictions, please upload the minimal anonymized data set necessary to replicate your study findings to a stable, public repository and provide us with the relevant URLs, DOIs, or accession numbers. Please see http://www.bmj.com/content/340/bmj.c181.long for guidelines on how to de-identify and prepare clinical data for publication. For a list of recommended repositories, please see https://journals.plos.org/plosone/s/recommended-repositories. You also have the option of uploading the data as Supporting Information files, but we would recommend depositing data directly to a data repository if possible.

Please update your Data Availability statement in the submission form accordingly.

Response : Thank you for pointing this out. All the data collected from the survey and used in the analysis has been presented within the manuscript as aggregates from which original dataset used in the study can be reconstructed, we will now change the data availability statement to following

In the online portal :

Do the authors confirm that all data underlying the findings described in their manuscript are fully available without restriction?

Our response : *Yes, all data are available fully without restriction*

Describe where the data may be found in full sentences. If you are copying our sample text, replace any instances of XXX with the appropriate details.

Our response: *All relevant data are within the manuscript*

f. Please update your submission to use the PLOS LaTeX template. The template and more information on our requirements for LaTeX submissions can be found at http://journals.plos.org/plosone/s/latex.

Response: Not applicable as this was not a LaTeX submission.

g . Please review your reference list to ensure that it is complete and correct. If you have cited papers that have been retracted, please include the rationale for doing so in the manuscript text, or remove these references and replace them with relevant current references. Any changes to the reference list should be mentioned in the rebuttal letter that accompanies your revised manuscript. If you need to cite a retracted article, indicate the article's retracted status in the References list and also include a citation and full reference for the retraction notice.

Response: The reference list has now been checked and updated accordingly. New references have now been included, based on the revision requests from the reviewers. The newly added citations are indicated in the manuscript on a track change mode for reference. .
